# Improved survival in patients with peritoneal metastases from colorectal cancer: a preliminary study

**DOI:** 10.1038/sj.bjc.6601586

**Published:** 2004-01-20

**Authors:** H Mahteme, J Hansson, Å Berglund, L Påhlman, B Glimelius, P Nygren, W Graf

**Affiliations:** 1 Department of Surgical Sciences, Akademiska Sjukhuset, Uppsala, Sweden; 2 Department of Oncology, Radiology and Clinical Immunology Akademiska Sjukhuset, Uppsala, Sweden

**Keywords:** colorectal cancer, peritoneal metastases, intraperitoneal chemotherapy

## Abstract

Patients with peritoneal or local metastases from colorectal cancer have a poor prognosis. However, aggressive treatments by debulking surgery and infusional intraperitoneal (i.p.) chemotherapy have been tried and appear to benefit selected patients. We assayed the effects of debulking surgery and i.p. chemotherapy with respect to survival and compared the results with matched control patients treated by intravenous (i.v.) chemotherapy. In all, 18 patients with peritoneal and/or local metastases from colorectal adenocarcinoma underwent debulking surgery followed by 5-fluorouracil (5-FU) 550 mg m^−2^ day^−1^ i.p. and leucovorin (LV) 60 mg m^−2^ day^−1^ i.v. The chemotherapy was started the day after surgery and was given daily for 6 days and repeated monthly for totally eight courses. The control patients, matched for age, gender, performance status and metastatic site, were randomly selected from controlled clinical chemotherapy trials and treated with i.v. 5-FU+LV or i.v. methotrexate+5-FU+LV. There was no treatment-related mortality. The median survival among i.p. patients was 32 months compared to 14 months in the control group. In all, 11 patients who underwent macroscopically radical surgery had a longer survival than those who were not radically operated (*P*=0.02). These results indicate that patients with peritoneal metastases and/or locally advanced cancers but without distant metastases may benefit from cytoreductive surgery combined with i.p. chemotherapy.

Peritoneal or local metastasis from colorectal cancer implies a poor prognosis (Graf *et al*, 1991; Mahteme *et al*, 1996; Shepherd *et al*, 1997; Assersohn *et al*, 1999) and the treatment remains a challenging problem. Moreover, patients with peritoneal carcinomatosis often suffer from intestinal obstruction and nutritional deficit (van Ooijen *et al*, 1993; Mahteme *et al*, 1996). In the absence of more effective therapeutic options, systemic 5-fluorouracil (5-FU)-based chemotherapy, irinotecan or oxaliplatin is used in order to achieve a regression of the tumour and improved outcome (Ragnhammar *et al*, 2001; Glimelius, 2003). Previous studies have demonstrated that chemotherapy prolongs survival about 4–6 months compared with supportive care alone (Colorectal Cancer Collaborative Group, 2000). The median survival time in patients with peritoneal carcinosis treated with modern chemotherapy is in the order of 6–12 months (de Gramont *et al*, 2000). Intraperitoneal (i.p.) 5-FU infusion has been suggested as an alternative route of administration with the purpose to expose peritoneal and local tumour remnants to high cytotoxic drug levels (Cunliffe and Sugarbaker, 1989), while only small amounts pass into systemic circulation. In recent years, there have been reports on i.p. chemotherapy treatment after cytoreductive surgery (Schellinx *et al*, 1996; Horsell *et al*, 1999; Culliford *et al*, 2001). A median survival of about 30 months was recently reported in patients treated with cytoreductive surgery plus i.p. chemotherapy (Elias *et al*, 2001).

The aim of this study was to explore the effects of cytoreductive surgery followed by repeated courses of i.p. chemotherapy with respect to feasibility, side effects and survival, and to compare with the results obtained using systemic chemotherapy.

## PATIENTS AND METHODS

### Patients characteristics

In all, 18 patients (nine women, nine men, mean age 54 years, range 31–74) were included in the study. The study was approved by the regional ethics committees. The protocol was set up in 1991 and the last patient was included in September 1999. The inclusion criteria were primary colorectal adenocarcinoma (colon 16, rectal 2), with local or peritoneal tumour deposits either resectable or suitable for debulking surgery, and without hepatic or other extra abdominal tumour growth as judged from laparotomy, chest X-ray and ultrasonography/CT scan, age <75 years and American Society of Anesthesiologists (ASA) classification grades 1–2. Informed consent was obtained from each patient. The diagnosis of the primary tumour and the metastases were verified histopathologically. One patient was not treated according to the protocol because of extensive irresectable peritoneal tumour growth. The remaining 17 patients were treated by either total macroscopic removal (11) or debulking (6) of the metastases followed by i.p. chemotherapy. In four patients, the diagnosis of local or peritoneal spread was carried out concomitant with the diagnosis of the primary tumour, and in the remaining 14 patients there was an interval of mean 19 (range 1–52) months between the diagnosis of the primary tumour and the local/peritoneal recurrence. A system for classification of local/peritoneal spread was set up based on which all patients could be classified: (a) predominant peritoneal growth±smaller local deposits; (b) predominant local growth±smaller peritoneal deposits; and (c) predominant abdominal wall growth±smaller local or peritoneal deposits. Two patients were classified in group a, six in group b and 10 in group c.

### Surgical treatment

The mean operating time was 3.7 h (range 0.9–6.7). The surgical procedure, the metastatic location and the treatments are detailed in Table 1

**Table 1:** Surgical procedures of the 18 patients in the i.p. group

**Preinclusion events**				**Events at inclusion**			**Postinclusive events**		
**Pat.**	**Primary tumour site**	**Surgical procedures at primary tumour surgery**	**Completing surgical events before inclusion**	**Metastatic site at inclusion**	**Surgical procedures at inclusion**	**Macroscopically radical**	**Remnant tumour location**	**Additional surgical tumour procedures**	**PAC reop.**
1	Colon	Colectomy, IRA	Rectal res. SBR × 2	Small bowel, abdominal wall, peritoneal	SBR, AWR, local excision	Yes		SBR	Yes
2	Colon	R. hemicolectomy		Ventricle, duodenum, small bowel × 2	ICR, duodenal resection, local excision, EC	Yes			Yes
3	Colon	Sigmoid resection, SOE		Peritoneal	Local excision, EC	Yes			No
4	Rectum	APR		Pelvic wall	Local excision	No	Pelvic	Scapula and costae resection	No
5	Colon	Ileocaecal resection		Ileocolic anastomosis, jejunum, abdominal wall	ICR, SBR, local excision	Yes		ICR × 2, SBR, AWR	Yes
6	Colon	Sigmoid resection, L. hemicolectomy, splenectomy		Abdominal wall, small bowel	AWR, SBR, local excision	Yes			No
7	Colon	Sigmoid resection R. hemicolectomy		Ileocolic anastomosis	ICR, local excision	Yes		R. hepatectomy, aorta resection, SBR	No
8	Colon	R. hemicolectomy		Ileocolic anastomosis, sigmoid, uterus, ovary, vagina, peritoneal	ICR, local excision, SOE, hysterectomy, EC	No	Small bowel (peritoneal) sigmoid colon (peritoneal)	SBR, rectosigmoid resection, ureterolysis	No
9	Colon	L. hemicolectomy	Colectomy+IRA, SOE	Abdominal wall, pelvic wall, peritoneal	AWR, local excision, EC	No	Pelvis	Local excision, EC	Yes
10	Colon	R. hemicolectomy		Pelvic wall, urinary bladder	Urinary bladder resection+urostoma, local excision	No	Pelvis		No
11	Colon	ICR		Abdominal wall, ileocolic anastomosis	R. hemicolectomy, AWR, Local excision	Yes			No
12	Colon	Sigmoid resection		Pelvic wall, rectum, small bowel, L.A. iliaca int, L. ureter	AR, iliaca int resection, local excision	Yes			No
13	Colon	R. hemicolectomy		Abdominal wall, small bowel, sigmoid colon	AWR, SBR, sigmoid resection	Yes		AWR	No
14	Colon	Ileocaecal resection		R. colon, small bowel, ovary, peritoneal	R. hemicolectomy, SOE, local excision, EC.	Yes			No
15	Rectum	APR, sigmoideostoma		Pelvic local, hepar, caecum	ICR, Liver resection, local excision	No	Pelvis		Yes
16	Colon	Sigmoid resection, pelvic wall resection		Ovary small bowel, colon transversum, peritoneal	Transversum resection, SBR, local excision, EC.	Yes			No
17	Colon	Sigmoid resection Hartmann resection, SBR		Peritoneal, locally advanced	Local excision	No	Peritoneal	Rectosigmoid anastomosis	No
18	Colon	R. hemicolectomy		Peritoneal, locally advanced	None	No, No PAC	Peritoneal, locally advanced		

AR=anterior resection; SOE=salpingo ofoor ectomy; APR=anterior perineal resection; AWR=abdominal wall resection; EC=electric cautery; ICR=ileocaecal resection; IRA=ileorectal anastomosis; PAC=port-A-cath; SBR=small bowel resection.

. At the end of surgery, a PORT-A-CATH (No. 21-2000-04, SIMS deltec, Inc., St Paul, MN, USA) was placed subcutaneously just above the periost of the lower ribs and a catheter was tunnelled through the abdominal wall and directed towards the principal tumour site. Finally, a drainage no. 18 was placed in the abdominal cavity. The drainage was plugged while the chemotherapy was given, but opened for drainage of peritoneal fluid for 1–2 h just before the next i.p. infusion. This drainage was removed at the end of the first treatment course.

### Intraperitoneal chemotherapy

The i.p. chemotherapy was started the day after surgery. 5-Fluorouracil was given i.p. (550 mg m^−2^ day^−1^) dissolved in 500 ml saline 0.9%. At 60 min after the start of the i.p. infusion, an i.v. infusion of leucovorin (LV) (60 mg m^−2^) was administered. The pharmacokinetical rationale for this sequential treatment is to obtain simultaneous tissue peak concentration of 5-FU and LV (Spears *et al*, 1989). The 5-FU dose was selected after a pilot study, showing that an i.p. 5-FU dose of 550 mg m^−2^ day^−1^ during 6 days was possible to give directly after major abdominal surgery without causing an increased risk for postoperative complications (Graf *et al*, 1994c). The chemotherapy treatment was given daily for 6 days with 4–6 weeks intervals. Any possible symptoms and side effects of the treatment were registered. Before the second course of treatment, a single photon emission computed tomography (SPECT) (General Electric, GE Maxxus, Milwaukee, WI) (Technetium-labelled albumin (^99^Tc^m^ Albures) at volume of 500 ml) was performed to judge the potential distribution of the drug in the abdominal cavity. The distribution of the drug was calculated using a computer-based measurement (FBP, Nuclear Diagnostics AB, Stockholm, Sweden). After the fourth course, a clinical evaluation was carried out. Another run of four courses was given if the patients responded well, tolerated treatment and had no clinical signs of tumour progression. After the eighth course, the patients were evaluated by clinical examination and CT scans/MRI or a second look. In two patients, the choice of the i.p. chemotherapy courses after the first one was based on *in vitro* assessment of chemotherapy-resistance test (Csoka *et al*, 1995). In these patients, i.p. cisplatin and i.p. irinotecan was given, respectively. The i.p. treatment was given as an outpatient procedure, except the first course, which was given directly after surgery.

### Control group

In all, 18 patients (nine women, nine men, mean age 56 years, range 36–69) treated for advanced colorectal adenocarcinoma within the Nordic chemotherapy trials (Nordic Gastrointestinal Tumour Adjuvant Therapy Group, 1992; Glimelius, 1993; Glimelius *et al*, 1998) were randomly selected as a reference group. The selection was made without any knowledge of survival. The selection criteria were: (1) resected primary colorectal adenocarcinoma (colon 15, rectal 3); (2) local or peritoneal tumour deposits; (3) no lymphatic, hepatic or extra abdominal tumour growth; (4) Karnofsky performance status >80 (mean 90, range 80–100); (5) treated by intravenous (i.v.) chemotherapy (eight patients received MFL and 10 received FLv). Furthermore, the control group was matched according to age and gender. The metastatic sites in this group were as follows: peritoneal (5), local (9) and peritoneal and local (4).

### Statistical methods

Survival curves were constructed according to the Kaplan–Mayer method and differences were analysed with the log-rank test. Differences in proportions were evaluated with Fisher exact test. A *P*-value below 0.05 was considered statistically significant.

## RESULTS

### Treatment effect

The median number of i.p. chemotherapy courses was 3 (1–8). Four patients had pain during or immediately after the i.p. infusion; however, none of the patients terminated the planned treatment because of infusion-related pain. Leakage from the drain site was noted in one patient. Two patients suffered repetitively from nausea and vomiting during the i.p. treatment period, and transient neutropenia was noted in one patient. Of the patients, 13 terminated the planned treatment prematurely, seven of them because of catheter-related problems (local catheter infection (1), improper position (1), obstruction (5)), ileus 1, liver metastases 1, decline in general status 1 and two patients refused further treatment. Single photon emission computed tomography studies showed a median abdominal cavity distribution volume of 2896 ml (range 32–11 557). In one patient, treatment was withdrawn after SPECT because of the lack of widespread distribution in abdominal cavity. In five patients the PORT-A-CATH was reoperated. There was no mortality related to surgery or to the i.p. treatment. In one patient who was not treated with cytoreductive surgery, no i.p. chemotherapy was administered.

### Survival

**Figure 1:**
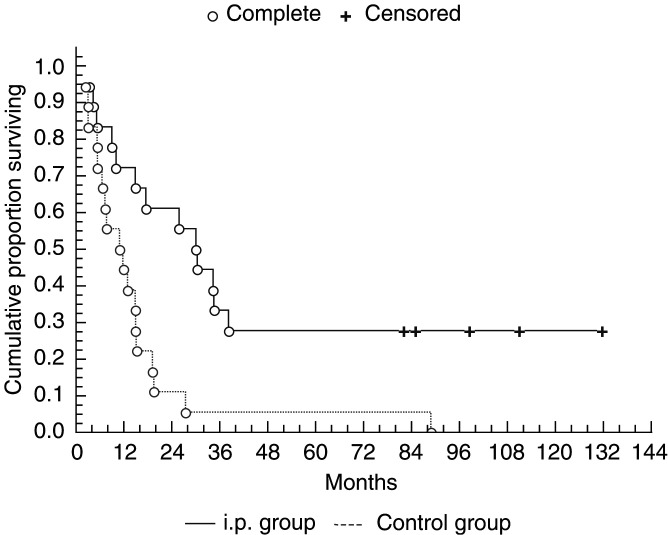
Cumulative proportion surviving (Kaplan–Meier).

**Figure 2:**
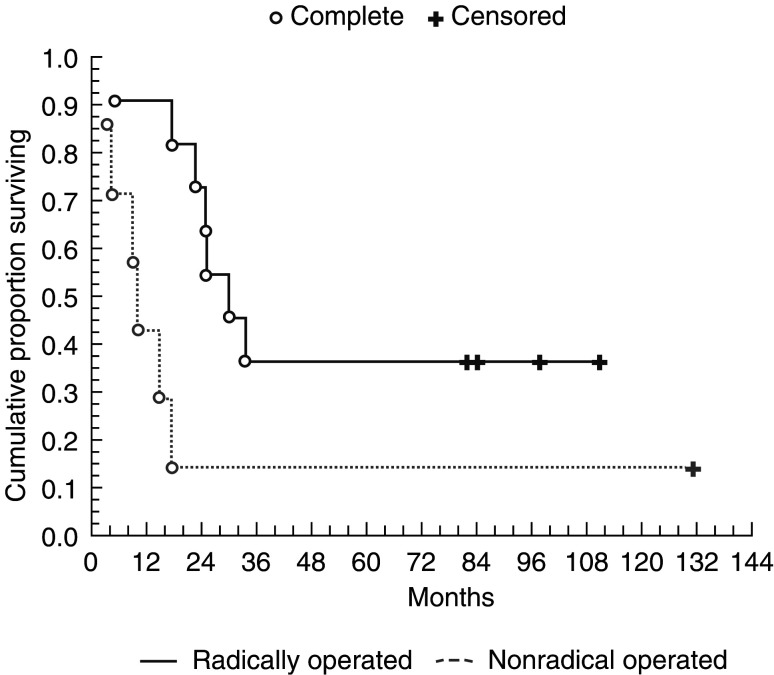
Cumulative proportion surviving (Kaplan–Meier).

The median survival in i.p. patients was 32 months (95% confidence interval (CI) 22.2–62.6 months), whereas in the i.v. control group it was 14 months (95% CI 5.6–24.9 months), (*P*=0.01, Figure 1

). A 2 and 5 years survival in i.p. patients were 60 and 28%, whereas corresponding values in the i.v. control group were 10 and 5%. In all, 11 patients who were considered macroscopically tumour free after the tumour reduction procedure had a longer survival (34.5 months, 95% CI 28.7–75.7) than those who did not undergo macroscopically radical surgery (10 months, 95% CI −15.7 to 70.0), (*P*=0.02, Figure 2

). Five patients in whom radical surgery could be performed are still alive (median 8.3 years, range 6.8–9.1) after surgery. One patient who underwent radical surgery survived only 4 months. One patient who was considered not to be macroscopicallly tumor free after the tumor reduction procedure is still alive and has survived 10.8 years. In total, 10 patients in whom radical surgery was not performed survived median 13 months (range 3 months–10.8 years).

## DISCUSSION

Our experience, with treatment of peritoneal colorectal metastases, is promising. We believe patients without hematogenous metastases (e.g. liver, lung, etc) from colorectal cancer might have a survival benefit if cytoreductive surgery is combined with i.p. chemotherapy. Furthermore, a complete remission of the disease is possible for an extended period of time. It seems that a macroscopically radical tumour resection has an impact on survival.

This series is not a prospective-randomised study, and a selection of patients may of course have influenced the results. However, in an attempt to compare the locoregional treatment to standard i.v. chemotherapy, we used historical controls. The two combinations (MFL and FLv) of chemotherapy, both based on biochemical modulation of 5-FU, were equally effective with respect to survival and response rates in one trial (Glimelius *et al*, 1998). It is therefore reasonable to consider these two combinations as equal and these patients thus received ‘golden standard’ chemotherapy during their treatment period. However, the more recently developed combination regimen (de Gramont *et al*, 2000; Douillard *et al*, 2000; Saltz *et al*, 2000) are even more effective than those used in the Nordic chemotherapy trials.

The relative importance of the i.p. chemotherapy cannot be properly assessed in the present study and further studies are needed to clarify a possible contribution of locoregional chemotherapy to the treatment effect. A possible benefit of a repeated regional treatment has been suggested since the end of 1960s (Long *et al*, 1969) and several reports have been published since then (Sugarbaker *et al*, 1996; Stephens *et al*, 1999; Cavaliere *et al*, 2000; Elias *et al*, 2001). One of the major problems is the nonuniform distribution of the chemotherapy to tumour deposits within the abdominal cavity. The SPECTs can be valuable to analyse the drug distribution in the abdominal cavity. If there are several adhesions, the labelled albumin will accumulate only in a limited space and chemotherapy may not reach all possible metastatic sites. To prevent postoperative adhesions and an obliterated abdomen, an early start of i.p. infusion, that is, immediately after surgery or at the latest the first postoperative day may be important.

One of the concerns of i.p. chemotherapy is anastomotic dehiscence. A study in humans indicated that it is possible to give the present regimen a day after surgery without suppressing the collagen accumulation too much (Graf *et al*, 1994a, 1994b). In addition, an experimental study showed an impaired healing after i.p. 5-FU, but when folinic acid was added, no further deterioration occurred (Graf *et al*, 1992). However, this problem and other chemotherapy-related toxicities have been investigated in several clinical studies and this form of administration has not been associated with an increased complication rate (Graf *et al*, 1994c; Vaillant *et al*, 2000).

The antitumoral effect of chemotherapy is believed to be enhanced by hyperthermia (41–42°C), possibly through an increase in cell membrane permeability, alteration of active drug transport, a change in cell metabolism and a decreased interstitial fluid pressure (Hahn and Shiu, 1983; Leunig *et al*, 1992; Kong *et al*, 2000). Moreover, recent clinical studies have shown promising results (Beaujard *et al*, 2000; Cavaliere *et al*, 2000). However, there is a lack of consensus about the optimal target temperature and a finding of increased morbidity and mortality when a cytoreduction procedure has been followed by hyperthermic i.p. chemotherapy (Jacquet *et al*, 1996) that warrant further studies. To optimise the i.p. treatment, choosing the appropriate chemotherapy is crucial. An important obstacle to successful treatment of solid tumours is the resistance to cytotoxic drugs (Wright *et al*, 1998; Germann, 2000; Keppler *et al*, 2000). In this context, the drug resistance examination is a potential valuable tool (Csoka *et al*, 1994, 1995).

In summary, a survival benefit can be achieved with cytoreductive surgery followed by repeated courses of i.p. chemotherapy. A complete remission of the disease is possible for an extended period of time. However, a longer period of follow-up is needed to establish if definite cure is possible for this category of patients and a randomised study is necessary to prove the value of this approach definitely.
